# Chufa Drink: Potential in Developing a New Plant-Based Fermented Dessert

**DOI:** 10.3390/foods10123010

**Published:** 2021-12-05

**Authors:** Sanne Kjærulf Madsen, Elisabeth Thule Thulesen, Mohammad Amin Mohammadifar, Claus Heiner Bang-Berthelsen

**Affiliations:** National Food Institute, Technical University of Denmark, 2800 Kongens Lyngby, Denmark; skma@food.dtu.dk (S.K.M.); elisabeththule@gmail.com (E.T.T.); moamo@food.dtu.dk (M.A.M.)

**Keywords:** *Cyperus esculentus*, lactic acid bacteria, fermentation, tiger nuts, dairy alternative, chufa drink, plant-based, cold butter-milk soup, *Leuconostoc mesenteroides*

## Abstract

Plant-based foods with desirable texture and nutritional value have attracted considerable interest from consumers. In order to meet the growing demand for more sustainable and health-focused products, new sources for plant-based products are needed. In this study, we aimed to develop an innovative plant-based dessert based on the underutilized crop chufa tubers (*Cyperus esculentus)*. The chufa extract was fermented with plant-adapted lactic acid bacteria and formulated with the purpose of imitating the Danish summer dessert “cold butter-milk soup”. The effect of various bacterial fermentations and formulations on steady and oscillatory rheology, stability, dry matter, pH, and sugar profile of the product were studied and compared to a commercial cold buttermilk soup sample. A strain of *Leuconostoc mesenteroides* was found to create the most similar taste to a commercial sample. By adding lemon juice, sucrose, xanthan gum, and vanilla to the fermented chufa drink, the drink was found to mimic the pH, texture, acid profile, and stability of a commercial dairy-based sample, while containing a lower concentration of carbohydrates.

## 1. Introduction

In recent years, plant-based foods have attracted considerable interest due to the consumers’ increasing awareness of health and animal welfare. A growing climate consciousness among consumers has given rise to more concerns regarding the environmental footprint of food production. An increasing number of people, therefore, have transitioned to a flexitarian, vegetarian, or vegan diet [1,2,3]. Meat and dairy products are major contributors to climate change because the livestock sector accounts for 14.5% of all human-induced greenhouse gas emissions [4]. According to Poore and Nemecek [5], animal-based products provide only 37% of proteins and 18% of calories in our diets, but the sector uses around 83% of the world’s farmland and accounts for approximately 58% of food-related greenhouse gas emissions.

Grains, nuts, and legumes are examples of plant foods that are commonly used in plant-based protein products, plant-based dairy alternatives, or meat replacers [1,6]. By acquiring knowledge regarding less known, underutilized crops and examining their potential as ingredients in foods, further plant-based alternatives can be offered to consumers and thus facilitate the shift towards a more plant-based diet.

*Cyperus esculentus* is a crop that produces edible root tubers known as chufa (also named tiger nuts or earth almonds) [7]. *C. esculentus* is cultivated in the Spanish Mediterranean region, in parts of Africa, and in the southern United States [7,8,9], but is also known as an invasive weed in many parts of the world [10,11]. Research on its sustainability is, however, very limited, and there exists to the authors’ knowledge no research on its environmental impact as a cultivated crop.

In Spain, chufa tubers (CT) are used for the production of a popular non-fermented milk-like extract called “Horchata de chufa” (HDC). The milk-like beverage is produced by soaking dried CT in water, grinding the soaked tubers into a paste, and extracting the liquid phase by pressing this paste [12,13]. The flavor of the chufa drink is described as slightly sweet, nutty, and coconut-like [8].

According to the literature, yields of oil from CT can vary from 15.9 to 41.2% when using hexane solvent extraction [14,15]. However, not all the oil in CT is being extracted into the liquid phase in the conventional production of HDC. Sánchez-Zapata et al. [12] reported the oil content of the chufa-based drink to be 2.4–3.1% (on fresh weight basis), which corresponds to approximately 26% of the total dry matter of the drink. CT also have a high content of starch, ranging from 20.1 to 41.7% of dry matter [7]. Some portion of the starch is also extracted into the liquid phase in the production of HDC; it has been reported that the drink contains approximately 3% starch [16].

In addition, CT are a rich source of dietary fiber. A substantial proportion of the dietary fibers has been reported to be the insoluble fibers such as cellulose and lignin [7]. Therefore, in the production of chufa milk-like beverages, the majority of dietary fibers are not extracted with the liquid phase, but remains in the solid residue [12].

Linssen et al. [7] reported that CT have a protein content in the range of 1.5–9.3% (on dry weight basis). Sánchez-Zapata et al. [12] observed the protein content of CT to be approximately 5%, but only 0.91% on average in HDC (on fresh weight basis). Furthermore, CT and extracts of the tubers are a source of vitamins and minerals such as vitamin E, vitamin C, iron, calcium, phosphorus, magnesium, and zinc [17,18,19].

Coşkuner et al. [20] found that the total sugar content of CT is approximately 15.4%, with sucrose constituting the majority of sugars (~13%) of dry matter. However, Abdel-Ahker et al. reported an average of 19.5% total sugar content [21]. The total natural sugar content of HDC varies from 3.7 to 15%, depending on variety and process conditions [18,22]. See Table 1 for an overview of the compositions of CT and HDC.

Although CT are mainly used commercially for the production of the milk-substitute HDC, their potential in the production of plant-based fermented products with similarities to yoghurt has also been recognized. Due to the high concentration of available sugars, fermentation with lactic acid bacteria (LAB) could result in yoghurt-like flavors. Previous attempts have been made to ferment chufa beverages by the addition of starter cultures [23,24,25,26,27]. Akoma et al. [23] found that yoghurt produced from chufa drink with commercial starter culture was slightly less liked than yoghurts containing cow milk. Wakil et al. [24] found that chufa drink fermented with wild type LAB was generally more accepted if the starter culture was mixed, but the overall acceptability was mediocre (around 4.3 out of 8 for the most-liked samples). Rita E. Sanful [25] found that yoghurt made from cow milk mixed with chufa drink was rated higher in appearance, sourness, consistency, and aroma compared to yoghurts made only from cow milk or chufa drink. Agbaje et al. [26] found that fermentation of chufa drink increased the content of minerals and amino and fatty acids. Maduka et al. [27] found that adding ginger or garlic to LAB fermented chufa milk increased the nutrient stability during storage. These findings indicate that there could be a market for fermented chufa drink, possibly for customers who either cannot or do not prefer to consume cow milk.

Despite this interest, no fermented chufa-based products, to our knowledge, are available on the Danish market. Such products could otherwise be offered as gluten-free and dairy-free options for vegans, gluten or lactose intolerant consumers, or anyone who wish to switch to non-dairy fermented products. The majority of these attempts have been carried out by fermenting with commercial starter cultures, mainly *Lactobacillus delbrueckii* subsp. *bulgaricus* and *Streptococcus thermophilus*. These organisms have been isolated from milk and could be adapted to the degradation of sugars and proteins found in bovine milk as seen for milk-adapted *Lactococcus lactis* [28]. This could result in the poor degradation of complex plant polymers, as Bachmann found [28].

Long-term stability of the chufa drink has previously been problematic due to phase-separation, which occurs due to sedimentation of starch granules at the bottom and creaming of fat droplets at the top [16,29,30]. By the addition of stabilizers such as xanthan gum and potato protein, a more stable structure might be created. Xanthan gum is a polysaccharide that is being produced by the fermenting bacteria *Xanthomonas campestris*. This polysaccharide has both stabilizing and thickening properties and is stable over a wide pH range [31].

The aim of this work was to study the potential of chufa milk in the production of a plant-based substitute to the Danish summer dessert “koldskål”, translated as “cold buttermilk soup”. This fermented dessert is a sweet and chilled buttermilk soup, which is usually flavored with lemon and vanilla, and has a consistency that is slightly more fluid than that of stirred yoghurt. Different types of plant-based yoghurt products are already found on the market [32,33], but to the authors’ knowledge there currently exist no commercial plant-based substitutes to the aforementioned buttermilk soup, although previous products have existed (examples: “S’ommerskål”, Naturli, Denmark, and “Plantekoldskål”, Mill-life, Denmark). In this study, a chufa-based liquid extract was subjected to fermentation with different strains of lactic acid bacteria in an attempt to produce a product with characteristics similar to those of cold buttermilk soup. Additionally, it was investigated how the addition of xanthan gum and potato protein to the fermented chufa drink affects texture, stability, and other attributes of the final product.

## 2. Materials and Methods

### 2.1. Preparation of Chufa-Based Liquid Extract

**Figure 1:**
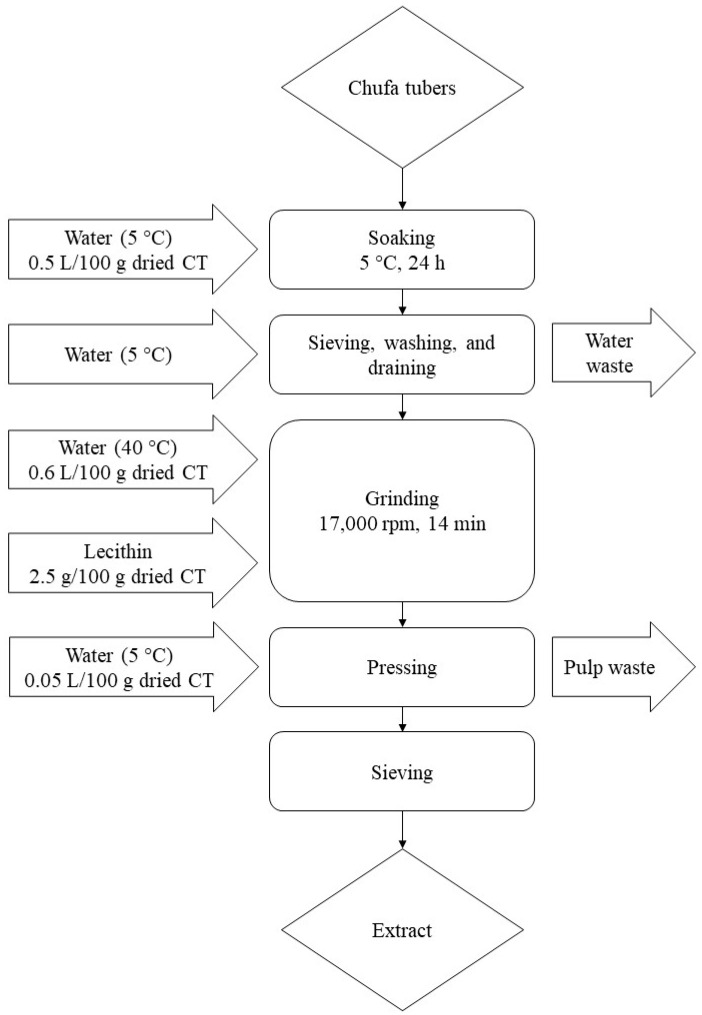
Flowchart of the process of producing the chufa-based liquid extract.

The process of producing a chufa-based liquid extract is illustrated in Figure 1. Dried CT were supplied from Nordic Chufa ApS, Denmark. Sunflower lecithin was added as an emulsifier in concentrations of 2.5 g/100 g dried CT during grinding to facilitate extraction of fat from CT. Soaking time, grinding time, amount and temperature of water for grinding, equipment for pressing, and pore size of the sieve were chosen based on previous experience.

For CT, 200 ± 1.0 g of CT were weighed in a bowl. One liter of cold, distilled water was added, and the bowl was covered by a plastic film. The tubers were soaked at 5 °C for 24 h. At the end of soaking, the water was discarded and the CT were rinsed with cold, distilled water and drained off in a sieve. The CT were first ground with a Bamix Gastro 200 W grinder (Bamix, Mettlen, Switzerland) in a plastic pitcher at 17,000 rpm for 8 min with 1200 mL of distilled water at 40 ± 3 °C. Subsequently, 5 ± 0.2 g of lecithin granulate (Biosym A/S, Ikast, Denmark) was added to the mixture, after which the grinding was continued for 6 min at 17,000 rpm. After grinding, the mixture was filtered with a Caffettiera French Press Coffee Maker containing a filter in stainless steel (Bodum, Triengen, Switzerland). One hundred milliliters of cold, distilled water was added to wash off leftovers of chufa pulp from the plastic pitcher, and this part was then added to the rest of the mixture in the press. The plunger of the press was pressed down by manual power until the liquid was extracted from the pulp. The liquid was then filtered through a mesh sieve in stainless steel (200 µm in pore size). The finished chufa drink was stored at 4 °C. An average production of 619.5 ± 10.1 mL per 100 g of CT was obtained. All measurements and tests performed on unfermented chufa drink were performed within 1–2 h of preparation.

### 2.2. Oil Content of Chufa-Based Liquid Extract

The oil content of the chufa drink was determined prior to fermentation and formulation using the extraction method by Bligh and Dyer [34]. Methanol, chloroform, and water was added to the chufa drink while stirring to extract the lipids. A supernatant containing methanol and water was separated from the lower layer, which consisted of chloroform and oil. The supernatant was removed, and the chloroform was evaporated to determine the oil content of the extract.

### 2.3. Fermentation of Chufa-Based Liquid Extract

Bacterial strains were obtained from the culture collection housed at the Technical University of Denmark (National Food Institute, Denmark). Qualified presumption of safety (QPS) listed strains belonging to the Lactobacillales order were selected for screening, and their genomes were analyzed for antibiotic resistance genes in silico. The genomes had been prepared in advance on a Nextseq 500 platform (Illumina) and assembled as described by Sørensen et al. [35]. Five strains previously used for acidifying plant bases were selected from different species, as shown in Table 2.

Preliminary experiments were conducted to find a suitable bacterial strain for the production of a fermented product based on chufa drink. The fermentation with the five strains were compared with one commercial starter culture (YFM, Chr. Hansen A/S, Denmark). The commercial starter culture consisted of a mix *of Lactobacillus delbrueckii* sp. *Bulgaricus* and *Streptococcus thermophilus*. This was done to assess whether a commercial strain would create a more complex and similar flavor to commercial cold buttermilk soup compared to a plant-isolated strain. Preparation of strains and inoculations were carried out under sterile conditions. Each strain from the laboratory collection was plated onto a de Man, Rogosa, Sharpe (MRS, Oxoid, UK) agar plate and incubated at 30 °C for 48 h. Single colonies were transferred to liquid MRS broth and incubated overnight at room temperature. Cells from the overnight cultures were harvested by centrifugation at 4000 rpm for 5 min at 5 °C. Supernatants were discarded and pellets were re-suspended in 40 mL sterile 0.9% NaCl. The centrifugation and re-suspension step was repeated once. Pellets were then centrifuged again at 4000 rpm for 5 min at 5 °C. Supernatants were discarded and pellets were re-suspended in 10 mL 0.9% sterile NaCl. The commercial culture YFM was prepared by adding freeze-dried pellets into 10 mL of sterile 0.9% NaCl until the solution became cloudy. Optical density (OD) of all the suspended bacterial solutions was measured at UV 600 nm on a UV3100PC (VWR, Radnor, PA, USA) spectrophotometer. The chufa drink was inoculated with overnight cultures and the commercial culture to a final OD of 0.01 and 0.1, respectively. The difference in OD was necessary in order to make up for the interference from the cryoprotectant used for the YFM culture. Cell counts were performed to determine the 10x increase of OD for the YFM culture. Samples with overnight cultures were then incubated at 30 °C and samples with the commercial culture at 40 °C (optimal temperature as instructed by Chr. Hansen A/S, Hørsholm, Denmark) in a thermostatically controlled water bath for 18 h. The pH of samples was monitored using an iCinac analyzer (AMS Alliance, Frépillon, France). After fermentation, the carbohydrates, organic acids, and alcohols were measured as described by Madsen et al. [37] for fermented and unfermented chufa drink, and a commercial buttermilk sample. An Ultimate HPLC (Dionex, Sunnyvale, CA, USA) system equipped with an Aminex HPX-87H column (Bio-Rad, Hercules, CA, USA) and coupled with a Shodex RI-101 refractive index detector (Showa Denko K.K., Tokyo, Japan) was used for the analysis. Results were calculated using the Chromeleon 7 software (Thermo Fisher Scientific, Waltham, MA, USA).

The taste and aroma of the samples were evaluated based on a simple sensory analysis. Non-trained, volunteering participants were asked to rate the samples from “least liked” to “most liked”. Comments on the taste, mouth-feel, and smell were recorded. The analysis was conducted as a blind-test. A score from 0 to 5 was awarded to each strain, with 0 being the “least liked” for each participant and 5 being the “most liked”. The strain with the highest average score was selected for further use to produce the formulated product. To mimic the pH of a commercial sample (pH 4.30), it was decided to adjust the duration of fermentation from 18 to 15 h. All following experiments with the selected strain were conducted in biological triplicates and carried out according to the procedure described in this section, with the only change being the pH being measured at 0 and 15 h post inoculation with an Excellent Line pH meter (Winlab, Germany) instead of the iCinac analyzer.

### 2.4. Formulation of Chufa-Based Product

Three samples of fermented chufa drink and one sample of non-fermented chufa drink was formulated in an attempt to mimic the Danish dessert cold buttermilk soup. The products were prepared in different varieties, as presented in Table 3. Potato protein (Protafy, KMC, Brande, Denmark) was added at a concentration of 1.5% (*w/v*) to two samples before fermentation to investigate its effect on structure development during fermentation. An amount of 0.4% (*w/w*) xanthan gum was added as thickener and stabilizer to three samples; 0.05% (*w/w*) vanilla beans, 2% (*w/w*) fresh lemon juice, and 6% (*w/w*) white sugar (sucrose) were added to all samples at the end of fermentation. Before analysis, samples were homogenized with a T 25 digital Ultra-Turrax disperser (IKA, Staufen, Germany) for 1 min at 17,000 rpm and kept refrigerated at 5 °C for one hour. A non-formulated, fermented sample of the chufa drink was included as control and a commercial, store-bought buttermilk soup (Løgismose, Broby, Denmark) sample was included as reference.

### 2.5. PH Measurements and Dry Matter Content

A 780 pH m (Metrohm, Herisau, Switzerland) was used for monitoring the pH of samples after formulation. Measurements were performed on biological triplicates. The dry matter content of samples was determined gravimetrically after the drying of samples in an oven at 105 °C (FD53, WTC Binder, Tuttlingen, Germany).

### 2.6. Rheology

Rheology of samples was studied to evaluate their flow behavior and structure. Measurements were conducted using a DHR-2 Rheometer equipped with a Peltier Concentric Cylinder Temperature System with an accuracy of ±0.01 °C (TA Instruments, New Castle, DE, USA). The concentric cylinder was configured with a Conical DIN Rotor and a Standard Concentric Cylinder Cup (TA Instruments, New Castle, DE, USA). Results were analyzed in the Trios software version 5.1.1.46572 (TA Instruments, New Castle, DE, USA). All experiments were conducted at 5 °C. Measurements were performed in triplicate.

Samples were subjected to a flow sweep test to investigate the correlations between shear stress and shear strain rate. The test was carried out with a shear rate increasing from 1 to 400 s^−1^, with 5 points measured per decade. Data for the different samples were compared using the Herschel–Bulkley model (τ = τ_y_ + m$\dot{\gamma }$^n^), where “τ” is the shear stress, “τ_y_” is the yield stress, “m” is the Herschel–Bulkly consistency coefficient, “$\dot{\gamma }$” is the shear strain rate, and “n” is the Herschel–Bulkly flow behavior index.

A strain sweep test was carried out to determine the yield point, τ_y_ (Limiting values of linear viscoelactic region in terms of shear stress, and the flow point, τ_f_, stress in the internal structure is ruptured to such an extent causing the material to flow (G′ = G″), of the samples. The test was carried out at a fixed frequency of 1.0 Hz and strain values ranging from 0.05 to 400%, with 5 points measured per decade.

A frequency sweep test was conducted to investigate the samples’ structure behavior. The test was carried out at a fixed strain of 0.2% and frequencies of 0.1–20 Hz. Data for the different samples were compared using the Power-Law model (G’ = a ω ^b^), where “G’” is the storage modulus, “a” indicates the structural strength of the sample, “b” indicates the type of structure, and “ω” is the angular frequency (s^−1^).

### 2.7. Stability Test

A Turbiscan Tower (Formulaction, France) was employed to quantitatively determine the stability of the samples. The analysis builds on the principle that a light source (λ = 880 nm) is moving across the sample. Transmission (T%) and backscattering (BS%) of the light source is monitored by a detector. The Turbiscan Tower scans the entire sample from the top, middle, and bottom, and repeats the scan of the sample at different time intervals, thus providing information on different instability phenomena such as creaming and sedimentation over a defined period of time [38]. The instrument reports the stability with a Turbiscan Stability Index (TSI). Calculation of TSI is automatically calculated based on an integrated algorithm that gives the cumulative sum of the T and BS variation of the whole sample, by Equation (1).

(1) $$SI\left(t\right)=\frac{1}{N_{h}}\overset{t_{max}}{\underset{t_{i=1}}{\sum }}\overset{z_{max}}{\underset{Z_{i}=z_{min}}{\sum }}\left|BST\left(t_{i},z_{i}\right)-BST\left(t_{i-1},z_{i}\right)\right|$$

This formula explains that, over the measurement of time ($t_{max}$) and within a given scan zone ($z_{min}$ and $z_{max}$), a given backscatter of transmission signal *BST* is measured, which will be *BS* if *T* < 0.2%, and else it will be *T.* $N_{h}$ is the height positions measured over time inside the scan zone $\left(\frac{z_{max}-z_{min}}{∆h}\right)$.

A 20 mL sample was added to flat-bottom cylindrical glass vials and placed in the Turbiscan Tower for 1 day, 3 h, and 10 min. Samples were scanned as follows: 20 scans every 3 min and 9 s for the first hour and 10 min, then 31 scans every 10 min until 6 h after start, and finally 21 scans once an hour until the end of the program. The analysis was carried out at 5 °C. Measurements were performed in triplicate. Signals were monitored and analyzed using TowerSoft software version 1.4.0.4.

### 2.8. Statistical Analysis

The analysis of variance (ANOVA) and Tukey–Kramer post-hoc test was carried out on the rheological data using the software JMP^®^ Pro, Version 15 (SAS Institute Inc., Cary, NC, USA). All tests were conducted in triplicate, except for tests on samples of FP1.5, which were carried out in duplicate due to experimental errors. All results were expressed by the mean value ± standard deviation. Results were added to rheology tables to express differences in values between groups. For pH, dry matter, and stability tests, the ANOVA and Tukey–Kramer post-hoc tests were carried out using GraphPad Prism 9.2.0. All tests were conducted in triplicate. For analysis on the stability test, results for each triplicate were grouped and plotted over time. A two-way ANOVA test was performed due to the size of the data set.

## 3. Results and Discussion

### 3.1. Oil Content of Chufa-Based Liquid Extract and Commercial Cold Buttermilk Soup

Oil content of the extract from the chufa tubers was measured prior to fermentation to examine whether an oil content similar to that of the commercial cold buttermilk soup could be obtained. The oil content of the commercial cold buttermilk soup was stated by the manufacturer to be 2.5%. When adding 2.5 g lecithin/100 g chufa tubers during the process of grinding, a total oil content of 2.8 ± 0.1% was achieved in the chufa-based extract. This similar amount of oil was desired because fat component can increase the mouthfeel and likability of the fermented chufa drink to mimic that of the commercial sample [39,40,41].

### 3.2. Sugar Profile of Chufa-Based Formulations and Commercial Cold Buttermilk Soup

To find a candidate strain for chufa fermentation, five strains of different lactic acid bacteria species and one commercial starter culture for milk were selected. Results of the HPLC analysis of the fermented and unfermented drink, and a commercial buttermilk sample, can be seen in Table 4.

The YFM-fermented product contained the most glucose after fermentation and had degraded the least galactose and cellubiose. It had a high citric acid content, only surpassed by DK130, and barely produced succinic acid, acetic acid, and ethanol. The YFM culture had the highest concentration of fermentable sugars left after fermentation, a total of 33.45 g/L. As explained by Bachmann [28], a bacterial culture that has been adapted to milk fermentation might perform poorly in plant bases due to graduate loss of the genes necessary for degradation.

The heterofermentative strains DK71 and DK93 were the most efficient at degrading the sucrose and galactose and produced high amounts of mannitol and acetic acid. Interestingly, the DK103 strain of *P. pentosaceus* showed heterofermentative behavior, producing mannitol, acetic acid, and ethanol in concentrations similar to DK71. A previous study by Tetlow et al. demonstrated similar experience with *P. pentosaceus*, where ethanol and acetic acid was produced in unexpectedly high levels [42]. A glucose peak was detected for DK71, DK93, and YFM after fermentation, suggesting they were degrading sucrose faster than they used glucose. DK130 and DK293 showed more homofermentative results, with the two highest concentrations of lactic acid, and low concentrations of mannitol, ethanol, and acetic acid. Citric acid was consumed by DK93 and DK103 and was produced for all other strains. Production of succinic acid and lactic acid was lowest for DK71 and the commercial strain. All strains produced ethanol in low amounts. The lowest pH was measured for DK71, although the highest concentration of acids was found for DK130. YFM, which had the highest pH, also had the pH closest to the commercial product.

Compared with the commercial buttermilk sample, the fermented samples were low in sugars and had lower concentrations of lactic acid. On the other hand, even though the cold buttermilk soup has added lemon juice, no citric acid was measured.

The outcome of the sensory analysis is shown in Table 5

The samples were ranked from worst to best in the following order: DK293, YFM, DK93, DK103, DK130, and DK71. DK71 was the only one to be described as having similarities to a commercial product and was selected for further use. Its acidity was found to be pleasant without having aftertastes. A possible explanation for this could be that the high amount of citric acid for DK71 gives it the fruity taste known from the commercial sample, and the high content of mannitol removes some of the bitter tastes, as previously seen [43,44]. The YFM and DK130 were found to have a sweet taste with little acidity, which could correlate with them having the two highest pH after fermentation. A high pH could indicate that the sugars in the medium have not been utilized by the bacteria. This is interesting because DK130 had the HPLC profile that matched the results for the commercial product the best: it had high sugar content, low acetic acid, mannitol, and ethanol, and the highest concentration of lactic acid. A previous study on beer [45] found that a lowered pH in some cases gives rise to off-flavors such as cardboard. Another study on milk-based yoghurt found a correlation between most sensory attributes and the pH [46]. There they found that flavors such as “creamy, buttery, sweet, milky, cottage cheese” all increased with an increase in pH, while flavors such as “astringent, acid, bitter, sour milk, lemon” all increased with a decrease in pH. Based on the HPLC results and the sensory test, DK71 was selected as the candidate strain for further experiments.

**Figure 2:**
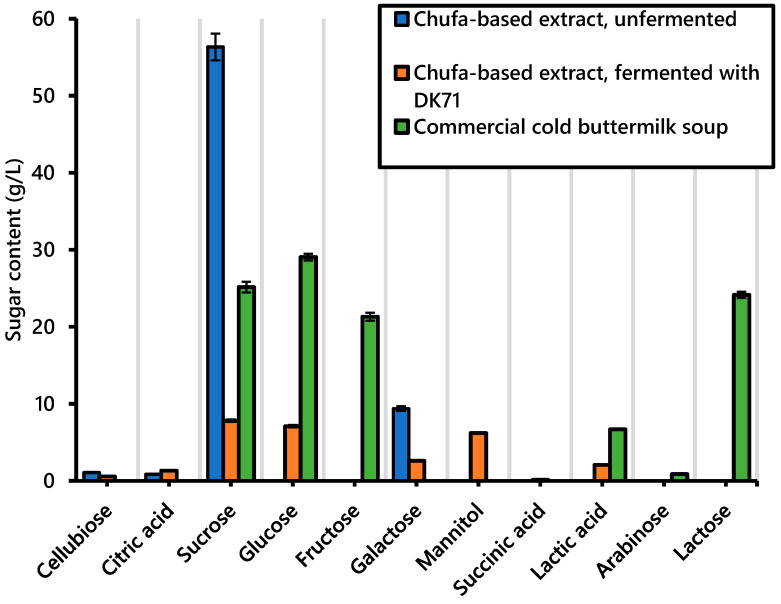
Sugar profile of unfermented chufa-based extract, fermented chufa-based extract, and a commercial cold buttermilk soup. Data are presented as mean values of triplicates. The error bars represent the standard deviation.

The free sugar profile of the chufa-based liquid extract was determined before and after it was subjected to fermentation with the strain DK71. Furthermore, the sugar profile of a commercial cold buttermilk was analyzed for comparison. As presented in Figure 2, sucrose constituted the majority of sugars (56.3 ± 1.7 g/L) in the unfermented chufa-based extract. This is consistent with previous findings [20]. The sucrose content of the fermented chufa-based extract was 7.8 ± 0.2 g/L, indicating that most of the sucrose had been converted to other compounds by the added lactic acid bacteria. Galactose concentration was measured at 9.4 ± 0.3 g/L in the unfermented extract. This is in good agreement with Marchyshyn et al. [47] and Parker et al. [48], who also observed that galactose composes a minor part of the sugars in chufa tubers. Much of the galactose had possibly been converted to other compounds by lactic acid bacteria because this sugar concentration was 2.6 ± 0.1 1 g/L after fermentation.

Glucose concentration was 7.1 ± 0.2 g/L in the fermented extract but was not detected in the unfermented extract. Because sucrose is a disaccharide consisting of one molecule of glucose and one molecule of fructose, the increased glucose concentration could be a result of the degradation of sucrose. Furthermore, some mannitol (6.2 ± 0.0 g/L) was detected in the fermented chufa-based extract. This was expected because mannitol is a sugar alcohol that can be produced as energy storage by heterofermentative lactic acid bacteria [49] and is in agreement with previous findings [37].

Lactic acid was produced in concentrations of 2.1 ± 0.1 g/L during fermentation of the chufa-based extract. By comparison, the amount of lactic acid in the commercial sample was 6.7 ± 0.1 g/L. Small amounts of acetic acid (1.0 ± 0.1 g/L) were also produced during fermentation. Similar amounts of acetic acid were measured in the commercial cold buttermilk soup (0.7 ± 0.0 g/L). Trace amounts of citric acid, cellubiose, and succinic acid were also detected in the unfermented and fermented chufa-based extracts.

In dairy fermentations, lactose is utilized by lactic acid bacteria to produce acidic compounds that contribute flavor and texture to the dairy product [50]. As reported previously in Table 3, unspecified lactic acid bacteria had been used to ferment the commercial product. However, not all lactose had been converted by the lactic acid bacteria, and some remained in the final product (24.1 ± 0.4 g/L). Lactose was expectedly not present in the chufa-based samples. Sucrose, glucose, and fructose in the commercial product were measured in the amounts of 25.2 ± 0.7 g/L, 29.0 ± 0.4 g/L, and 21.3 ± 0.5 g/L, respectively. It was stated by the manufacturer that syrup had been added to the product, but the type of syrup was not specified. It is assumed that the content of sucrose, glucose, and fructose originated from this syrup. As discussed above, the unfermented chufa-based extract had a high content of sucrose that would naturally contribute sweetness. Nevertheless, most of the sugars in the chufa-based extract were converted to other compounds by the added lactic acid bacteria during fermentation, resulting in a product with concentrations of sugars that were considerably low compared to the sugar concentrations of the commercial product. Therefore, sugar was added in the concentration of 6.0% to formulated samples (as shown in Table 3) to compensate for the loss of sugars and yield a product with a sweetness similar to that of the commercial cold buttermilk soup. In total, the carbohydrate content of the formulated fermented chufa extract was 8.17%, where it was 9.96% for the commercial sample.

### 3.3. Dry Matter and pH of Chufa-Based Formulations and Commercial Cold Buttermilk Soup

Dry matter content and pH of samples are presented in Table 6. The fermented product was designed to mimic the dry matter content and pH of the commercial sample so the products would be comparable when measuring rheology and stability. The pH of a non-fermented, non-formulated chufa sample was 6.60.

For all formulated samples, vanilla extract, lemon juice, and sucrose were added. The non-formulated sample (F) had a relatively low dry matter content (9.0 ± 0.0%) compared to the commercial sample of cold buttermilk soup (15.9 ± 0.1%). All the formulated samples, however, had a dry matter content that was similar to that of the commercial sample as it was in the range of 14.0–15.9%. The addition of 6.0% sugar to the samples was likely responsible for most of the increase in dry matter content of the formulated products. As presented in Table 6, pH of the commercial sample was 4.3 ± 0.0, whereas pH of the fermented chufa-based samples was in the range of 3.7–4.1. The fermented sample containing xanthan gum (FX0.4) had the lowest pH (3.7 ± 0.0), where acids from the fermentation and from the lemon juice contributed. Adding potato protein to the drink (FX0.4P1.5 and FP1.5) raised the pH of the samples to a slight extent (3.9 ± 0.0) and increased the dry matter to match the commercial sample. Adding xanthan gum and potato protein together did not make a difference to the pH compared with just adding potato protein. It is therefore likely that xanthan gum did not contribute significantly to changes in the pH. Proteins are known to have buffering capacities, which could explain the slight increase in pH noticed for the samples containing potato protein [51]. The commercial sample is reported by the producer to contain 3.2% protein. By adding 1.5% potato protein, the fermented chufa drink would have a more comparable protein content because chufa drink only has about 0.6–1.4% protein [12]. However, this could affect the taste and aroma of the drink negatively, as potato protein has been reported to have bitter off-flavors [52]. The fermented, non-formulated sample had a pH closest to the commercial sample (4.1 ± 0.0). However, it was not possible to maintain pH at this level after formulation with acidifying ingredients such as lemon juice. Although fermentation already contributed to lowering the pH of the chufa-based extract, it still contained lower amounts of lactic acid compared to the commercial product, as reported previously. The concentration of lemon juice in the commercial sample was 1.5% (see Table 3), but it was decided to add 2% lemon juice to the chufa-based samples to compensate for the lower concentration of lactic acid and enhance the acidic flavor of the product. This resulted in formulated products with pH values slightly lower than those of the commercial sample. In total, the carbohydrate content of the formulated fermented chufa extract FX0.4 was 8.17%, where it was 9.96% for the commercial sample.

### 3.4. Rheology of Chufa-Based Formulations and Commercial Cold Buttermilk Soup

Data obtained from the flow sweep tests were compared using the Herschel–Bulkley model and shown in Table 7.

As presented in Table 7, high values of R^2^ indicate that the model was a good fit for the data. The variable “m” in the Herschel–Bulkley model is the consistency coefficient, which is a measure of the samples’ resistance to flow. Higher “m” values indicate higher consistency/viscosity [53]. The three samples, NFX0.4, FX0.4, and FX0.4P1.5, exhibited a very similar consistency coefficient. The addition of 1.5% potato protein to the extract did not seem to have a significant impact on consistency either. As expected, samples containing the thickening agent xanthan gum displayed a thicker consistency than samples without this additive. The commercial sample had a consistency coefficient of 1570 ± 311 mPas^n^. As mentioned previously, fermentation of bovine milk leads to the formation of a gel-like network in the product, resulting in a higher consistency. Furthermore, egg yolk is added in the commercial sample to increase thickness. The low consistency coefficients of F and FP1.5 revealed that this type of structure development was not achievable in the chufa-based extract, and the addition of potato protein prior to fermentation did not have an impact on the extract’s consistency either. Only by adding xanthan gum was it possible to obtain a difference in consistency for the extract. The consistency coefficient of the commercial sample was, however, still approximately 1.6 times as high. It is probably feasible to obtain a consistency that is closer to that of the commercial product by reducing the concentration of added xanthan gum in the chufa-based extract.

The Herschel–Bulkley flow behavior value “n” indicates whether the sample have shear-thinning (n < 1) or shear-thickening (n > 1) [53]. There was no significant difference in the flow behavior index of NFX0.4, FX0.4, or FX0.4P1.5. These samples showed shear-thinning behavior because their n values was in the range of 0.47–0.49, indicating that the viscosity of the samples decreased when the shear rate increased. The commercial cold buttermilk soup also showed shear-thinning behavior because the n value of this sample was measured to be 0.44 ± 0.01. Although the n values of F and FP.15 were significantly different from each other, the samples had a flow behavior index close to 1. Thus, the shear stress of these samples was close to being linearly correlated to the shear strain rate. This indicates that these samples showed Newtonian fluid behavior and had viscosities that were almost independent of shear rate. Hence, without the addition of xanthan gum as thickener, the chufa-based extract had rheological properties similar to those of diluted solutions.

The yield stress “τ_y_” expresses the amount of stress that will cause the sample to deform plastically instead of elastically. In other words, the value of shear stress that is required before the sample undergoes permanent deformation [53]. The samples NFX0.4, FX0.4, and FX0.4P1.5 had a yield stress in the range of 3.45–3.91 Pa, and they were not significantly different from each other. Thus, the yield stress levels of the samples were dependent on whether the extract had been fermented or contained potato protein. The commercial cold buttermilk soup showed a yield stress of 2.40 ± 0.12 Pa, which is a little lower than that of the chufa-based samples containing xanthan gum. The yield stresses and n value of both FP1.5 and F were close to 0 and 1, respectively, which is expected of samples with Newtonian behavior.

Yield stress is not a material constant and may vary depending on the method of measurement. The yield stress value (τ_y_) and the flow point (τ_f_) for the samples were also determined by a strain sweep test. Results are presented in Table 8.

The yield point, τ_y_, is the point where the samples undergo permanent deformation. The flow point, τ_f_, is the value of shear stress at the crossover point (G′ = G′′) for the storage modulus (G′) and the loss modulus (G″), and it describes the shear stress required to make the sample flow [53]. As discussed above, the flow sweep test revealed that NFX0.4, FX0.4, and FX0.4P1.5 had the same level of consistency. However, the strain sweep test showed that the yield point, τ_y_, of the unfermented sample NFX0.4 differed significantly from the yield points of the fermented samples FX0.4 and FX0.4P1.5. It required a lower level of shear stress to break the structure of the fermented chufa-based samples compared to the unfermented sample, although these samples were formulated with the same concentration of xanthan gum. As presented in Table 6, fermentation resulted in a lower pH of samples compared to the pH of the unfermented sample. It is likely that the more acidic conditions of the fermented samples had led to less solubility of starch and more aggregation, thereby leading to a structure with more resistance towards destruction.

Only a small amount of shear stress (0.39 ± 0.02 Pa) was required before the commercial sample was permanently deformed. Unlike the three samples with xanthan gum, the commercial did not contain any thickening agents. This gives evidence that the addition of xanthan gum to the chufa-based extracts resulted in a structure with higher resistance to mechanical stress compared to the commercial. The yield points for the samples FP1.5 and F were close to 0. Because xanthan gum was not added to those samples, they demonstrated a fluid-like behavior, and a negligible amount of stress was required for the samples to yield. The flow points, τ_f_, of NFX0.4, FX0.4, and FX0.4P1.5 were in the range of 6.38–7.04 Pa. Less shear stress was required to make the commercial cold buttermilk soup flow as it showed a flow point of 2.81 ± 0.32 Pa. The samples FP1.5 and F had flow points close to 0, thus again showing a fluid-like behavior. The adjustment of yield stress would be important to mimic the mouth feel of a commercial sample. The data demonstrates that formulation with 0.4% xanthan gum increased the chufa-based samples’ resistance to flow to an extent that is was close to that of commercial samples.

Data obtained from the frequency sweep test are shown in Table 9.

The variable “b” gives information regarding the type of behavior; the closer b approaches 0, the more similar is the structure to a true gel. A high b-value relates to a more gel-like structure. The variable “a” describes the strength of the gel; the higher the value, the stronger the structure. R^2^ was higher than 0.98 for all samples, indicating that the model fitted well to the data. The b-values of the commercial and the samples containing xanthan gum were all in the same range (0.16–0.19) and did not differ significantly from each other. This implies that xanthan develops a similar network to that found in the commercial sample. In the absence of xanthan, the frequency sweep pattern of FP1.5 and F was similar to that of a dilute solution, with higher b-values of 0.25–0.30, which the results from the strain sweep also indicated.

The a-values of NFX0.4, FX0.4, and FX0.4P1.5 were in the range of 8.45–10.35 Pa. The commercial had the highest value (23.15 ± 5.21 Pa), which means that it had the highest structural strength. Interestingly, the commercial sample had a lower yield stress compared to the samples containing xanthan gum, as reported above. Although the commercial sample was shown to be the strongest gel, it was more sensitive to mechanical forces than those other samples. The chufa-based extracts containing xanthan gum, on the other hand, had weaker structures but required a higher amount of stress to start flowing. This is in agreement with the results found in the flow sweep tests.

### 3.5. Stability of Chufa-Based Formulations and Commercial Cold Buttermilk Soup

**Figure 3:**
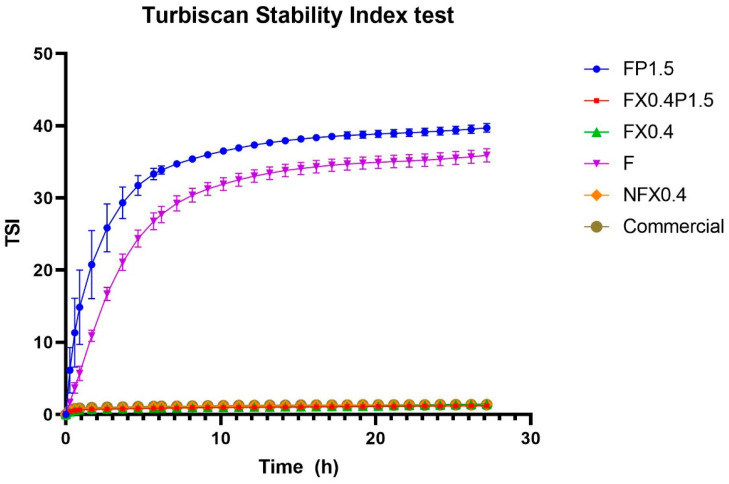
Turbiscan Stability Index. Measured over 27 h at 5 °C of non-fermented chufa drink with 0.4% xanthan gum (NFX0.4), fermented chufa drink with 0.4% xanthan gum (FX0.4), fermented chufa drink with 0.4% xanthan gum and 1.5% potato protein (FX0.4P1.5), fermented chufa drink with 1.5% potato protein (FP1.5), fermented, non-formulated chufa drink (F), and a commercial cold buttermilk soup. Data are presented as mean values of triplicates.

The global results from the Turbiscan Stability Index, measured over time for fermented and non-fermented chufa drink with different formulations, can be seen in Figure 3.

The results of the statistical analysis of the global TSI measurements can be seen in Table 10.

TSI values of samples were measured to determine the samples’ stability during storage at 5 °C for 27 h. TSI values lower than 1 indicate that the sample is stable and uniform, while TSI values higher than 1 indicate that the sample has undergone destabilization as described by the producer. The fermented, non-formulated sample had reached a TSI of 35.91 ± 0.92 by the end of the test. It is most likely that a phase-separation arising between the liquid phase and the heavier starch granules of the chufa drink was causing the non-formulated sample to destabilize during storage. The stability of three out of four formulated samples (NFX0.4, FX0.4, and FX0.4P1.5) was observed to be considerably improved compared to the non-formulated sample because they displayed a TSI value close to 1 during the storage period. By the end of the test, the TSI of NFX0.4, FX0.4, and FX0.4P1.5 had reached 1.39 ± 0.20, 1.46 ± 0.30, and 1.22 ± 0.12, respectively, which was comparable to the commercial sample at 1.37 ± 0.44. The statistical analysis further confirmed this by grouping the samples containing xanthan gum with the commercial sample, both for the maximum and for the mean TSI. This indicates that the samples were still as stable and uniform as the commercial sample after 27 h of storage. Xanthan gum is known to provide long-term stability to colloid systems by its ability to increase the viscosity of solutions at low shear rates [31]. Xanthan gum is thought to be responsible for suspending the starch molecules in the liquid solution, thereby increasing viscosity of the solution and avoiding sedimentation to occur. One sample, NFX0.4, was not fermented, but formulated identically to the fermented sample, FX0.4. The difference in TSI values of these samples was negligible and, therefore, the stability of the chufa drink is not considered to be influenced by fermentation. However, it cannot be ruled out that it was an effect of combining xanthan gum and acidic conditions that resulted in an improved stability of NFX0.4, FX0.4, and FX0.4P1.5. It is likely that the starch granules in the chufa drink were modified by acidification with citric acid from lemon juice. The glucose chains in amylopectin and amylose, which are the main constituents of starch, can be hydrolyzed by citric acid, causing the large starch granules to break down into shorter chains of glucose molecules [54]. These smaller molecules have lower molecular weights than those of the large starch granules and may not sediment as easily. This could be the reason that acidification with lemon juice contributed to an enhanced stability of the samples. The sample with 2% lemon juice, no xanthan gum, and 1.5% potato protein (FP1.5), however, still had a poor stability; TSI reached 39.7 after 27 h of storage. This value is comparable to the TSI value of the non-formulated control sample, which also exhibited poor stability.

## 4. Conclusions

An innovative plant-based dairy dessert was prepared by fermenting a chufa-based drink extract with selected lactic acid bacteria from the University’s in-house strain collection of plant, food, and environmentally collected isolates. A commercial starter culture, YFM, was used to compare conventional cow milk-fermenting strains with the plant isolates. Analysis of the sugar profile revealed that most of the sucrose in the chufa-based extract was utilized by the lactic acid bacteria during fermentation. The chufa-based extracts, especially when fermented, contained fewer simple carbohydrates than the commercial strain. The commercial strain was less effective in converting the sugars into acids and had the highest remaining sugar concentration after 18 h of fermentation. A simple sensory analysis revealed that most of the fermented chufa had unpleasant off-flavors, especially related to the taste of acetic acid. The commercial strain and the least acidifying of the plant-isolated strains were both described to have a flat, sweet taste. One strain, DK71, was described as having a pleasant taste with similarities to a commercial product and was selected for further use. Fermented and unfermented chufa-based extracts were subsequently formulated in an attempt to imitate the characteristics of the Danish summer dessert “cold buttermilk soup”. Using lecithin as an emulsifier during the process of grinding the chufa tubers resulted in a total oil content similar to that in a commercial product. The pH of the fermented, formulated samples was slightly lower compared to the pH of the commercial sample. Samples containing potato protein had a slight increase in pH compared to samples without. Rheological measurements showed that the chufa-based samples containing xanthan gum had higher viscosities than samples without this thickener. The addition of potato protein prior to fermentation or the fermentation itself did not have a significant impact on the samples’ viscosities. Dry matter contents of all the formulated samples were similar to that of the commercial product, while it was lower for the non-formulated sample. Both the chufa-based samples with xanthan gum and the commercial product with egg yolk had gel-like properties, but the chufa-based extracts were found to be slightly weaker gels than the commercial product. However, they had a higher resistance to mechanical stress compared to the commercial. Samples with xanthan gum were all shown to have an improved stability that was as good as the stability of the commercial sample, unlike chufa-based extracts without added xanthan gum. This indicates the need for adding ingredients with thickening and stabilizing effects to the fermented chufa-based drink to make a product with properties similar to those of the commercial cold buttermilk soup. Furthermore, the amount of carbohydrates in the formulated drink was lower than in the commercial.

To create a product with commercial potential, future work should focus on sensory analysis of the formulated product and elucidate whether the texture, flavor, and aroma of the fermented chufa-based drink can be further enhanced, along with shelf life of the formulated product compared to a commercial product. The nutritional profile of the formulated chufa drink compared to a commercial could also be of further interest.

## Figures and Tables

**Table 1:** Nutritional composition of chufa tubers (dry weight) and Horchata de chufa (wet weight).

Source	Oil (%)	Starch (%)	Protein (%)	Sugar (%)
Chufa tubers (dry weight)	15.9–41.2 [14,15]	20.1–41.7 [7]	1.5–9.3 [7]	15.4–19.5 [20,21]
Horchata de chufa (wet weight)	2.4–3.1 [12]	2.0–3.0 [12]	0.6–1.4 [12]	9.0–10.0 [18,22]

**Table 2:** Meta data for the five in-house strains. All strains were isolated from locations in Denmark.

ID Name	Organism	Origin	Isolation Site	Fermentation Type
DK71	*Leuconostoc mesenteroides*	Gooseberry	Rosmosevej, Blovstrød	Obligately heterofermentative ^1^
DK93	*Leuconostoc citreum*	Spinach	Rosmosevej, Blovstrød	Obligately heterofermentative ^1^
DK103	*Pediococcus pentosaceus*	Pumpkin	Rosmosevej, Blovstrød	Obligate homofermentative ^1^
DK130	*Lactococcus lactis*	Fava bean	Frilandsmuseet	Obligate homofermentative ^1^
DK293	*Lactoplantibacillus plantarum*	Wild Plant Sp.	Drangsholm castle	Facultative heterofermentative ^1^

^1^ Source: [36].

**Table 3:** Samples of chufa-based drink prepared with different formulations, compared to a commercial formulation. Potato protein is expressed as % *w/v*, and the remaining ingredients are expressed as % *w/w*.

Sample ID	Fermented	Formulation
NFX0.4	No	0.05% vanilla, 2% lemon juice, 6% sucrose, 0.4% xanthan gum
FX0.4	Yes	0.05% vanilla, 2% lemon juice, 6% sucrose, 0.4% xanthan gum
FX0.4P1.5	Yes	0.05% vanilla, 2% lemon juice, 6% sucrose, 0.4% xanthan gum, 1.5% potato protein
FP1.5	Yes	0.05% vanilla, 2% lemon juice, 6% sucrose, 1.5% potato protein
F	Yes	No formulation
Commercial *	Yes	Dairy milk, syrup, egg yolk, lemon juice, water, salt, vanilla, vanilla seeds, starter culture

* Formulation from the commercial sample was obtained from the producer.

**Table 4:** Sugar, acid, and alcohol concentrations after fermentation. Concentrations are in g/L.

Sample Name	DK71	DK93	DK103	DK130	DK293	YFM	Un-Fermented ^1^	Commercial Buttermilk ^1^
Sucrose	6.38	0.30	10.57	21.48	19.82	13.35	56.34	25.16
Glucose	6.82	0.26	n.d *	n.d	n.d	10.61	n.d	29.05
Galactose	1.89	0.26	1.92	5.20	4.65	5.64	9.38	n.d
Fructose	1.82	5.56	3.25	2.27	2.93	3.85	n.d	21.31
Lactose	n.d	n.d	n.d	n.d	n.d	n.d	n.d	24.15
Cellubiose	0.00	0.54	0.59	0.59	0.28	0.62	0.61	n.d
Citric acid	1.20	0.34	0.39	1.98	1.42	1.89	0.86	n.d
Succinic acid	0.13	0.31	0.22	0.15	0.16	0.04	n.d	n.d
Lactic acid	2.40	2.52	3.11	4.48	3.32	2.75	n.d	6.69
Acetic acid	1.17	1.66	1.39	0.33	0.50	0.28	n.d	0.68
Mannitol	7.85	9.24	7.38	1.34	2.22	5.64	n.d	n.d
Ethanol	0.54	0.18	0.45	0.24	0.24	0.11	n.d	n.d
Final pH	3.85	3.90	3.88	4.00	3.87	4.19	6.60	4.30

* n.d = not detected ^1^ average of 3 samples.

**Table 5:** Results of the sensory analysis. The average rating was ranked from least liked (0) to most liked (5).

Sample ID	Strain	Average Rating	Comments
1	DK93	2	Smells and taste sweet like dark bread. The sourness is stingy, like acetic acid. As thin as milk.
2	YFM	1	Too sweet, has an aftertaste like cereal, no sourness, thin like milk. Smells fruity and sweet.
3	DK103	3	Sense of bubbling on the tongue, has a dusty aftertaste. A bit thicker than milk. Leaves a film on the tongue.
4	DK71	5	Sour in a fruity way, like citric acid. Similar to the sourness of a commercial product. A bit more texture than the other samples. Smells like fermented milk with a sweet undertone.
5	DK293	0	Sweet taste but with unpleasant acidity. Taste like butyric and acidic acid. Has a thicker texture than other samples. Leaves a bad aftertaste.
6	DK130	4	Overly sweet. Taste and smell like chufa drink without the coconut flavors. Thin as milk. Very flat taste and smell. A bit acidic, like citric and lactic acid.

**Table 6:** Dry matter content and pH of fermented, non-fermented, and commercial samples. Data are presented as mean values of triplicates ± standard deviation.

Sample ID *	Fermented	Dry Matter (%)	pH
NFX0.4	No	14.00 ± 0.10 ^b^	4.60 ± 0.01 ^b^
FX0.4	Yes	14.70 ± 0.10 ^c^	3.70 ± 0.01 ^c^
FX0.4P1.5	Yes	15.90 ± 0.04 ^a^	3.90 ± 0.02 ^a^
FP1.5	Yes	15.10 ± 0.10 ^d^	3.90 ± 0.02 ^a^
F	Yes	9.00 ± 0.03 ^e^	4.10 ± 0.01 ^d^
Commercial	Yes	15.90 ± 0.05 ^a^	4.30 ± 0.01 ^e^

* Non-fermented chufa drink with 0.4% xanthan gum (NFX0.4), fermented chufa drink with 0.4% xanthan gum (FX0.4), fermented chufa drink with 0.4% xanthan gum and 1.5% potato protein (FX0.4P1.5), fermented chufa drink with 1.5% potato protein (FP1.5), fermented, non-formulated chufa drink (F), store-bought sample (Commercial). Values (a, b, c, d, e) for each column not connected by the same letters are significantly different.

**Table 7:** Herschel–Bulkley model parameters for samples. Data are presented as mean values of triplicates ± standard deviation. The third sample of FP1.5 was excluded. Values for each column not connected by the same letters are significantly different.

Herschel–Bulkley (τ = τ_y_ + m$\dot{\gamma }$^n^)				
Sample ID	m (mPa.s^n^)	n *	τ_y_ (Pa)	R^2^
NFX0.4	815± 18 ^b^	0.47 ± 0.01 ^b^	3.45 ± 0.07 ^a^	0.99 ± 0.00
FX0.4	850 ± 9 ^b^	0.48 ± 0.00 ^b^	3.91 ± 0.04 ^a^	0.99 ± 0.00
FX0.4P1.5	857 ± 69 ^b^	0.49 ± 0.01 ^b^	3.65 ± 0.26 ^a^	0.99 ± 0.00
FP1.5	4 ± 1 ^c^	0.99 ± 0.03 ^a^	0.01 ± 0.00 ^b^	0.99 ± 0.00
F	5 ± 1 ^b^	0.98 ± 0.02 ^a^	0.02 ± 0.00 ^b^	0.99 ± 0.00
Commercial	1570 ± 311 ^a^	0.38 ± 0.01 ^c^	2.40 ± 0.12 ^a^	0.99 ± 0.00

* The flow index is dimensionless.

**Table 8:** Strain sweep test values for samples. Data are presented as mean values of triplicates ± standard deviation. The third sample of FP1.5 was excluded. Means values not connected by same letters are significantly different.

Strain Sweep (G′)		
Sample ID	τ_y_ (Pa)	τ_f_ (Pa)
NFX0.4	2.36 ± 0.15 ^b^	6.38 ± 0.11 ^a^
FX0.4	2.96 ± 0.16 ^a^	7.04 ± 0.25 ^a^
FX0.4P1.5	3.00 ± 0.1 ^a^	6.75 ± 0.33 ^a^
FP1.5	0.004 ± 0.001 ^d^	0.01 ± 0.00 ^b^
F	0.01 ± 0.00 ^d^	0.03 ± 0.01 ^b^
Commercial	0.39 ± 0.02 ^c^	2.81 ± 0. 32 ^c^

**Table 9:** Frequency sweep test values for samples. Data are presented as mean values of triplicates ± standard deviation. The third sample of FP1.5 was excluded.

Frequency Sweep (G’)			
Sample ID	a (Pa s^n^)	b *	R^2^
NFX0.4	10.35 ± 0.52 ^a^	0.19 ± 0.01 ^a^	0.99 ± 0.01
FX0.4	8.82 ± 0.35 ^a^	0.18 ± 0.00 ^a^	0.99 ± 0.00
FX0.4P1.5	8.45 ± 0.8 ^a^	0.18 ± 0.01 ^a^	0.99 ± 0.00
FP1.5	0.07 ± 0.03 ^b^	0.30 ± 0.15 ^b^	0.98 ± 0.02
F	0.16 ± 0.03 ^b^	0.25 ± 0.03 ^ab^	0.99 ± 0.03
Commercial	23.15 ± 5.21 ^c^	0.16 ± 0.01 ^a^	0.99 ± 0.00

* The flow behavior index is dimensionless. Values (a, b, c) for each column not connected by the same letters are significantly different.

**Table 10:** Minimum, maximum, and mean values TSI for each sample. Means values not connected by same letters are significantly different.

Sample	Minimum TSI *	Maximum TSI	Mean TSI
FP1.5	0.00 ^a^	39.70 ± 0.59 ^b^	25.76 ± 11.83 ^b^
FX0.4P1.5	0.00 ^a^	1.22 ± 0.12 ^a^	0.78 ± 0.26 ^a^
FX0.4	0.00 ^a^	1.46 ± 0.30 ^a^	0.83 ± 0.30 ^a^
F	0.00 ^a^	35.91 ± 0.92 ^c^	19.29 ± 12.39 ^c^
NFX0.4	0.00 ^a^	1.40 ± 0.15 ^a^	0.91 ± 0.29 ^a^
Commercial	0.00 ^a^	1.37 ± 0.44 ^a^	1.01 ± 0.28 ^a^

* Values (a, b, c) for each column not connected by the same letters are significantly different.

## Data Availability

Genomes of plant-isolated strains can be found at NCBI under the BioProject number PRJNA785875.
